# La-Induced Phase Transformation and Band Structure Modulation of Bi_2_O_3_ for Enhanced Visible-Light Photocatalytic Degradation of Rhodamine B

**DOI:** 10.3390/nano16161025

**Published:** 2026-08-18

**Authors:** Qiuqin Wang, Yongkui Wang, Chao Feng, Xiaoqi Jin, Jinlong Ge, Cuishuan Xu

**Affiliations:** School of Materials and Chemical Engineering, Anhui Provincial Engineering Laboratory of Silicon Based Materials, Engineering Technology Research Center of Silicon-Based Materials (Anhui), Functional Powder Materials Laboratory of Bengbu City, Bengbu University, Bengbu 233030, China; wangqiuqin000@163.com (Q.W.); fchg042@163.com (C.F.); 13167526736@163.com (X.J.); jinlongge2005@126.com (J.G.); xucuishuan@yeah.net (C.X.)

**Keywords:** La doping, Bi_2_O_3_, phase transformation, band structure modulation, photocatalytic degradation

## Abstract

Using bismuth oxide (Bi_2_O_3_) as the matrix and employing a doping modification strategy to introduce the rare-earth element La, this study prepared La/Bi_2_O_3_ visible-light-responsive photocatalysts with different doping ratios. The research systematically investigated the regulation mechanisms of La doping on the material’s phase structure, microstructure, band structure characteristics, and visible-light photocatalytic performance. The results indicate that an appropriate amount of La^3+^ equivalently substitutes Bi^3+^ in the lattice, inducing the complete transformation of pure α-Bi_2_O_3_ into the tetragonal β-Bi_2_O_3_ phase while maintaining the integrity of the crystal framework. Meanwhile, the modulation of the local electronic structure caused by La^3+^ substitution effectively narrows the bandgap width and broadens the visible-light response range; it also acts as an electron trap to significantly suppress the recombination of photo-generated electron–hole pairs, thereby enhancing charge transport efficiency. Visible-light catalytic degradation experiments confirmed that 4% La/Bi_2_O_3_ exhibits the optimal degradation kinetics for RhB, achieving a 72.88% degradation rate of Rhodamine B within 60 min of visible-light irradiation. The first-order reaction rate constant was 23 times that of pure Bi_2_O_3_, and the material demonstrated good stability under repeated cycles. Radical trapping experiments indicated that the order of contribution of active species was ·O_2_^−^ > h^+^ > ·OH, with the superoxide radical (·O_2_^−^) being the dominant active species. This study confirms that appropriate lattice doping with La can synergistically optimize the structure and optoelectronic properties of Bi_2_O_3_, providing experimental evidence and theoretical references for the rational design of highly efficient and stable visible-light-responsive Bi_2_O_3_-based photocatalytic materials.

## 1. Introduction

Amid the rapid pace of industrialization and urbanization, water bodies continue to absorb large quantities of organic dyes, antibiotics, and various persistent organic pollutants [1,2,3], which not only disrupt the ecological balance of water ecosystems but also pose a significant threat to human health [4]. Photocatalytic oxidation technology is a green and highly efficient advanced water treatment process that utilizes light energy to trigger redox reactions under ambient temperature and pressure conditions, thereby achieving the complete mineralization and decomposition of organic pollutants in water. Mu et al. [5] prepared ZnWO_4_/g-C_3_N_4_S heterojunction nanofibers, which utilize oxygen vacancies to facilitate the deprotonation of water molecules and provide protons, and employ the built-in electric field within the heterojunction to effectively separate photo-generated charge carriers. The photocatalytic nitrogen fixation rate reached 144.5 μmol·L^−1^·min^−1^·g^−1^, which is 19.5 times that of pure g-C_3_N_4_. After five cycles, the performance remained at 90.8%, demonstrating excellent stability. The BiVO_4_/BiOI/ACF composite photocatalytic material prepared by Zhang et al. [6] achieved a phenol degradation efficiency of 91.1% within 180 min under visible light through adsorption–photocatalytic synergy. Moreover, the material is in macroscopic bulk form, making it easy to recover. Luan et al. [7] constructed a Ho_2_FeSbO_7_/Bi_0.5_Yb_0.5_O_1.5_ Z-type heterojunction photocatalyst, which achieved a 99.82% removal efficiency for ciprofloxacin within 140 min of visible-light irradiation, with a total organic carbon (TOC) removal rate of 97.63%, confirming that photocatalytic technology can efficiently degrade antibiotic-based organic pollutants. Gao et al. [8] used P25TiO_2_ as a photocatalyst to degrade five typical pharmaceuticals and personal care products in medical wastewater under natural sunlight. The results showed that the degradation rates for all five pollutants reached 100%, with a total organic carbon (TOC) removal rate exceeding 95%, achieving near-complete degradation. The above studies demonstrate that photocatalytic technology offers advantages such as mild reaction conditions, the ability to utilize sunlight, and the capacity to efficiently degrade and even mineralize organic pollutants, all without generating secondary pollution. It thus shows broad application prospects in the field of wastewater treatment.

It is currently one of the core technological approaches for addressing water pollution and improving water quality. Among various semiconductor photocatalytic material systems, bismuth trioxide (Bi_2_O_3_) has emerged as a key research material in the field of photocatalysis due to its suitable bandgap of 2.6–2.8 eV, excellent chemical stability, and good visible-light absorption properties. Mane et al. [9] pointed out in their review that Bi_2_O_3_ has six crystalline structures (α, β, γ, δ, ε, ω), among which α-Bi_2_O_3_ is the most stable monoclinic phase at room temperature, while β-Bi_2_O_3_ (tetragonal phase) exhibits superior photocatalytic activity under visible light due to its narrower bandgap (approximately 2.24–2.27 eV). Jangra et al. [10] prepared a LaAlO_3_/Bi_2_O_3_ nanocomposite photocatalyst; by constructing a Type I heterojunction, they effectively promoted the separation of photo-generated charge carriers, achieving a 94% degradation efficiency for methylene blue within 160 min. Fahim et al. [11] investigated the effects of Cd doping on the electronic structure and optical properties of cubic Bi_2_O_3_ using first-principles calculations and found that doping can effectively tune the bandgap and enhance light absorption efficiency. Furthermore, Bi_2_O_3_ offers advantages such as low toxicity, low cost, and diverse synthesis methods, and has been widely applied in fields such as photocatalytic degradation of organic pollutants, antibacterial applications, and supercapacitors. However, pure phase Bi_2_O_3_ has significant application limitations that severely restrict its photocatalytic performance and practical engineering value: the electron–hole pairs generated by light excitation in the material tend to recombine rapidly [12,13,14], resulting in a low quantum yield; simultaneously, its visible-light absorption performance is limited [15,16,17], and the particles in powder form are highly prone to agglomeration [18,19,20], leading to poor dispersion uniformity. These multiple defects collectively hinder the full realization of its photocatalytic degradation efficiency.

It is worth noting that Bi_2_O_3_ is a typical polymorphic semiconductor material with a rich variety of crystal structures; among these, the monoclinic α-Bi_2_O_3_ and tetragonal β-Bi_2_O_3_ phases are currently the two most extensively studied and widely applied crystal forms [21,22,23]. Different crystal phases of Bi_2_O_3_ exhibit significant differences in key properties such as band structure, carrier mobility, and the number and distribution of active surface sites, which directly result in the two crystal phases demonstrating distinctly different photocatalytic degradation capabilities [24]. Existing research has confirmed that β-Bi_2_O_3_ possesses a narrower bandgap and superior specific surface area. Compared to α- Bi_2_O_3_, β-Bi_2_O_3_ exhibits more outstanding visible-light utilization efficiency and catalytic activity [25]. Based on these characteristics, inducing a phase transformation from α-Bi_2_O_3_ to β-Bi_2_O_3_ through appropriate preparation and control methods has become a reliable and efficient modification strategy for optimizing and enhancing the overall photocatalytic performance of Bi_2_O_3_-based materials.

Numerous studies have confirmed that rare-earth doping can effectively modulate the electronic structure of metal oxide semiconductors while creating defect sites within the crystal lattice. Kiramani et al. [26] reported the co-doping of La^3+^ and Yb^3+^ at the A sites in SrTiO_3_. The study found that dual-rare-earth doping narrowed the bandgap from 3.21 eV to 3.00 eV and effectively suppressed the recombination of photo-generated carriers by introducing oxygen vacancies and lattice distortion, thereby increasing the degradation efficiency of methylene blue from 61.04% to 73.27%. Bagyalakshmi et al. [27] demonstrated that Ag-assisted substitution of metal ions such as La, Zn, and Cd can regulate the oxygen vacancy concentration and surface defect states in cobalt oxides, thereby influencing their visible-light catalytic performance. Yang et al. [28] found that La^3+^ doping-induced gradient oxygen vacancies (a 270% increase), combined with Ti^3+^/Ti^4+^ redox pairs, narrowed the bandgap of BiFeO_3_ from 2.13 eV to 1.79 eV, significantly extending the carrier lifetime and achieving a Rhodamine B degradation efficiency of 98.9%. In et al. [29] prepared Ce-doped In_2_O_3_ nanofibers via electrospinning and found that Ce^3+^ doping effectively narrows the bandgap and enhances photocatalytic activity. Based on the aforementioned rare-earth doping modulation strategies, this work selected La for lattice doping modification of Bi_2_O_3_. Since the ionic radii of La^3+^ (1.03 Å) and Bi^3+^ (1.03 Å) are similar, equivalent ion substitution is easily achieved, resulting in relatively mild lattice distortion. The introduction of La doping is expected to generate oxygen vacancies [30], modulate the positions of band edges [31], refine grain size, and induce a morphological transformation from a dense bulk structure to a three-dimensional mesoporous nanosheet network [32], thereby enhancing the material’s photocatalytic activity [33,34]. Nevertheless, systematic investigations are currently lacking; for example, the critical La doping ratio required to induce the α→β phase transition, the intrinsic relationship between doping concentration evolution and lattice defect evolution, and the intrinsic relationship among doping level, microstructural evolution, and photocatalytic activity remain unclear.

This work systematically investigated the effects of different La doping levels on the phase structure, microstructure, band structure, and photo-generated carrier dynamics of Bi_2_O_3_, with the aim of elucidating the intrinsic relationship between La doping levels, structural evolution, and photocatalytic activity, and determining the optimal doping ratio. For the first time, a threshold effect was revealed whereby a 1% La doping level is sufficient to induce the complete transformation of α-Bi_2_O_3_ into β-Bi_2_O_3_, providing a clear reference for low-dose rare-earth-induced phase engineering. The mechanism by which La doping enhances photocatalytic performance through the synergistic effects of phase regulation, band structure optimization, morphological restructuring, and improved charge separation efficiency was elucidated. A 4% La doping ratio was determined to be optimal, yielding a reaction rate constant 23 times that of pure Bi_2_O_3_. Based on this, a structure–property relationship was established between the La doping concentration, the material’s multiscale structure, and its catalytic performance. This study provides experimental evidence and theoretical guidance for the rational design of highly efficient and stable visible-light-responsive Bi_2_O_3_-based photocatalytic materials.

## 2. Materials and Methods

### 2.1. Materials

All chemicals were used as received without further purification. Bismuth(III) nitrate pentahydrate (Bi(NO)_3_·5H_2_O, AR) was purchased from Shanghai Macklin Biochemical Company, Limited (Shanghai, China). Glycine (C_2_H_5_NO_2_, AR) and ethanol (C_2_H_6_O, AR) were obtained from Sinopharm Chemical Reagent Co., Ltd. (Shanghai, China). Lanthanum (III) nitrate hydrate (La(NO_3_)_3_·xH_2_O, AR) was supplied by Shanghai Titan Technology Company, Limited (Shanghai, China). Rhodamine B (C_28_H_31_ClN_2_O_3_, AR) was purchased from Adamas-beta. Deionized water was prepared in the laboratory.

### 2.2. Synthesis of Materials

Using Bi(NO)_3_·5H_2_O as the bismuth source and C_2_H_5_NO_2_ as the fuel [35], Bi(NO)_3_·5H_2_O was dissolved in C_2_H_5_NO_2_ at a molar ratio of 1:3. Weigh 3.64 g of g Bi(NO)_3_·5H_2_O and 1.68 g of C_2_H_5_NO_2_ into 30 mL of water. Under magnetic stirring, a stoichiometric amount of La(NO_3_)_3_·xH_2_O was added to the mixture. After stirring the mixture for 30 min, it was transferred to a crucible. When the tube furnace reaches 500 °C, insert the crucible, calcine for 10 min, then remove it to obtain the sample. At high temperatures, the combustion of the fuel releases a large amount of heat, causing Bi(NO_3_)_3_ to decompose and form solid products consisting of bismuth oxide and rare-earth composite oxides. The catalyst doped with La at a concentration of x% (molar ratio of La to bismuth), is designated as x% RE/Bi_2_O_3_ (x = 0, 1, 2, 3, 4, 5). Photos of some of the powder products can be found in Supplementary Figure S1.

### 2.3. Characterizations

The crystal structure of the samples was characterized using an X-ray diffractometer (XRD, Rigaku SmartLab SE, Rigaku Corporation, Tokyo, Japan) equipped with a Cu Kα radiation source (λ = 1.5406 Å), operating at a voltage of 40 kV and a current of 40 mA. Diffraction patterns were acquired in the 2θ range from 10° to 80° with a scan increment of 0.02°. A field-emission scanning electron microscope (SEM, Nova NanoSEM 450, FEI Company, Hillsboro, OR, USA) was used to observe the microstructure of the samples at an acceleration voltage of 20.00 kV. The elemental composition of the surface was analyzed using an X-ray photoelectron spectrometer (XPS, ESCALAB 250Xi, Thermo Fisher Scientific, Waltham, MA, USA) with a monochromatic Al Kα radiation source; all binding energies were corrected using the exogenous carbon C 1s peak (284.8 eV). Ultraviolet-visible diffuse reflectance spectroscopy (UV-Vis DRS) was measured using a UV-Vis spectrophotometer (U3900, Hitachi High-Technologies Corporation, Tokyo, Japan) equipped with an integrating sphere accessory, with BaSO_4_ as the reference, over a scanning range of 400–650 nm at a scanning rate of 300 nm/min. Nitrogen adsorption–desorption isotherms were measured at 77 K using a fully automated specific surface area and pore size analyzer (TriStar II 3020, Micromeritics Instrument Corporation, Norcross, GA, USA). Prior to testing, all samples were degassed at 160 °C for 6 h, and the specific surface area was calculated using the Brunauer–Emmett–Teller (BET) method. Phosphorescence (PL) spectra were recorded at room temperature using a fluorescence spectrometer (F-4600, Hitachi, Japan), with the excitation wavelength set to 280 nm and the emission spectrum acquisition range set to 340–390 nm. Fourier transform infrared (FTIR) spectra were measured using a spectrometer (IS10, Thermo Fisher Scientific, USA) via the KBr pellet method, with a scanning range of 4000–500 cm^−1^. In the photocatalytic experiments, Rhodamine B (RhB) was used as the target compound for degradation, and its degradation performance was evaluated under visible-light irradiation. The reaction solution was monitored at a constant scanning rate within the wavelength range of 450–650 nm.

### 2.4. Photocatalytic Oxidation Experiment of Cyclohexane

Using RhB as the target degradation product, the photocatalytic activity of the prepared Bi_2_O_3_ samples was evaluated under visible-light irradiation. The experimental setup is shown in Figure S2. The photocatalytic reaction was conducted in a quartz reactor (100 mL volume) equipped with a circulating water cooling jacket. The light source was a 500 W xenon lamp (λ ≥ 420 nm, Guangzhou Yuye Light Source Manufacturing, Guangzhou, China), the irradiance was measured by a radiometer to be 520 mW/cm^2^. All reactions were conducted at room temperature (25 ± 2 °C) to avoid interference from thermal effects. The specific procedure is as follows: Disperse 0.2 g of catalyst in 50 mL of a 10 mg/L RhB solution and place it in the quartz reactor. First, the mixture was magnetically stirred in the dark (at 500 rpm) for 30 min to allow the system to reach adsorption–desorption equilibrium; subsequently, 5 mL of the suspension was taken as the initial concentration sample (denoted as C_0_). The light source was then turned on, and samples of the reaction mixture were collected every 10 min, for a total of 6 collections (total illumination time of 60 min), to monitor the kinetic changes during the degradation process. The collected samples were immediately centrifuged at 10,000 rpm for 5 min to remove catalyst particles. The absorbance of the supernatant at the maximum absorption wavelength of RhB (554 nm) was measured using a Hitachi U-3900 UV-Vis spectrophotometer, and the residual concentration was calculated using a standard curve.

### 2.5. Testing of Photoelectrochemical Properties

Photoelectrochemical characterization was performed using an electrochemical workstation (CHI760E, Shanghai Chenhua Instrument Company, Limited, Shanghai, China). The tests included electrochemical impedance spectroscopy (EIS), transient photocurrent response, and Mott–Schottky curves. The experimental setup and electrode connections are shown in Figure S3. The entire testing procedure employed a standard three-electrode system: a platinum wire (0.5 mm in diameter, 99.99% purity) served as the counter electrode, a saturated potassium chloride system with a calomel electrode as the reference electrode, and a 0.5 mol/L aqueous Na_2_SO_4_ solution as the electrolyte, with a volume of 100 mL. The working electrode preparation procedure is as follows: Weigh 10 mg of the sample to be tested, disperse it in 2 mL of deionized water, and sonicate for 10 min to obtain a uniform slurry. The slurry was evenly coated onto the surface of a pretreated, clean ITO conductive glass substrate (1.0 cm × 2.0 cm, thickness 1.1 mm), with an effective coated area of 1.0 cm × 1.0 cm. It was then dried at a constant temperature of 60 °C for 6 h to ensure that the sample film adhered firmly. Transient photocurrent testing was performed using an intermittent illumination mode with a 500 W xenon lamp as the light source. The light source was cyclically turned on and off, with both the illumination and dark periods set to 30 s each. A bias voltage of 0.6 V (vs. SCE) was applied, and the dynamic changes in current density over time were recorded simultaneously. Electrochemical impedance spectroscopy (EIS) testing was performed at open-circuit potential, with a frequency range of 10^−2^ to 10^5^ Hz and an AC amplitude of 5 mV. Mott–Schottky curve measurements were performed in the dark, with a frequency of 1000 Hz, a potential scan range of –1.3 to 1.3 V (vs. SCE), and a scan rate of 10 mV/s.

## 3. Results and Discussions

### 3.1. Crystalline Structure

**Figure 1:**
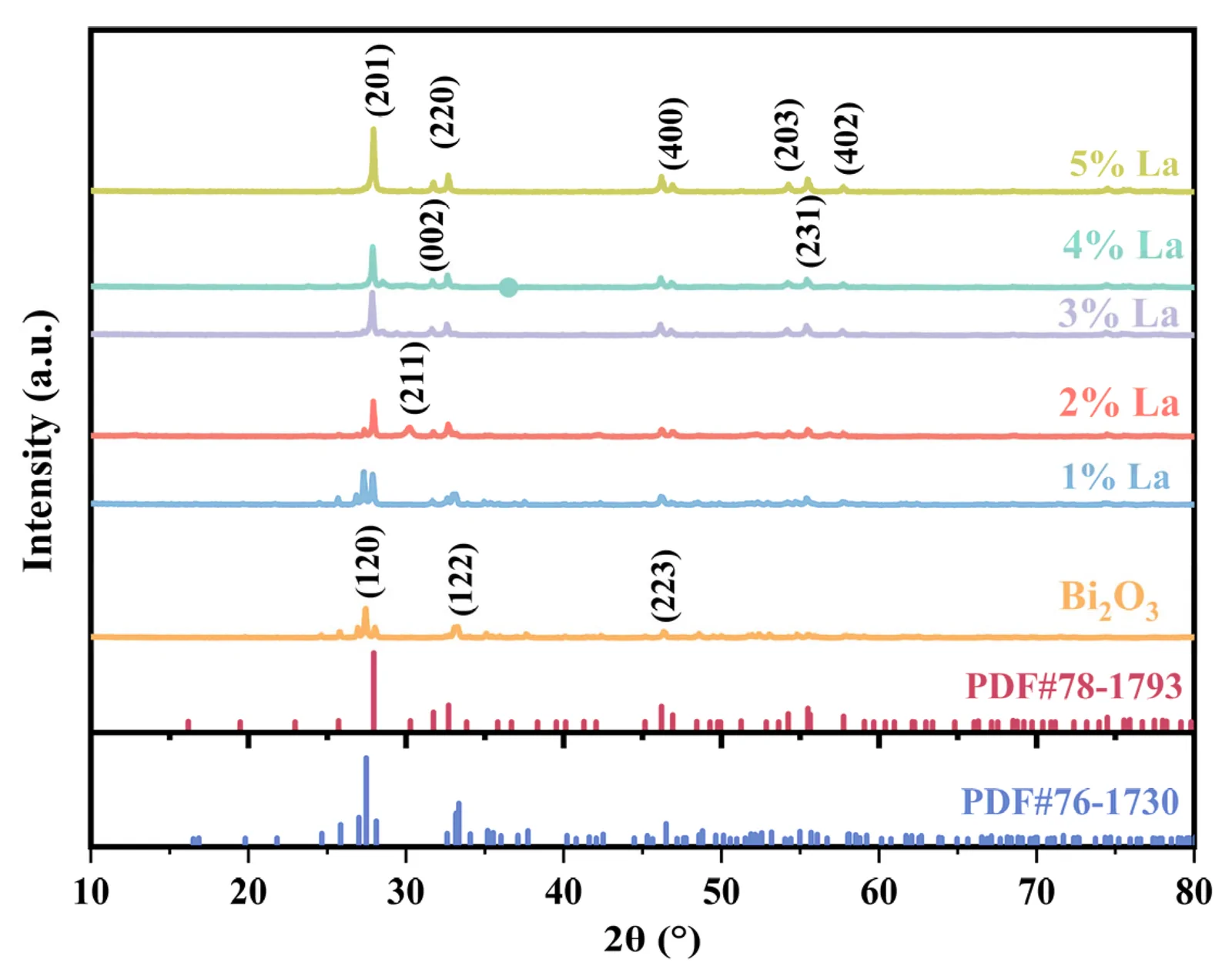
XRD patterns of Bi_2_O_3_ and La doped Bi_2_O_3_.

Analysis of the XRD patterns of Bi_2_O_3_ samples with different La doping levels (Figure 1) shows that the characteristic diffraction peaks of the pure Bi_2_O_3_ sample perfectly match the standard card (PDF#76-1730) for the monoclinic phase α-Bi_2_O_3_. The strongest diffraction peak at 2θ ≈ 27.5° corresponds to the (120) crystal plane of α-Bi_2_O_3_, with no impurity peaks present, indicating a pure α-Bi_2_O_3_ phase. The peaks are sharp, demonstrating excellent intrinsic crystallinity.

Upon the introduction of La, the main diffraction peak of the sample shifted from the (120) plane of the α phase (2θ = 27.5°) to the (201) plane of the tetragonal β-Bi_2_O_3_ standard card (PDF#78-1793) (2θ = 27.9°). At a La doping level of 1%, the diffraction peak of the β phase began to grow significantly, indicating that even extremely low levels of La doping can induce a phase transformation in Bi_2_O_3_. Within the La doping range of 2–5%, the diffraction peaks of all samples matched the tetragonal β-Bi_2_O_3_ phase well, and no characteristic diffraction signals of La_2_O_3_ or other impurity phases were observed, proving that La^3+^ successfully substituted Bi^3+^ in the lattice to form a single-phase substitution solid solution [36]. Since the ionic radii of La^3+^ (~1.03 Å) and Bi^3+^ (~1.03 Å, CN = 6) are highly similar, the equivalent substitution did not cause significant lattice distortion. Therefore, the positions of the characteristic diffraction peaks in the doped samples did not shift noticeably [37,38]. Furthermore, as the La doping level increased from 1% to 5%, the diffraction intensity of the main β-phase peak continued to rise (primarily due to the increase in β-phase content), and the half-width at half-maximum of the diffraction peak gradually narrowed while the peak shape became sharper, indicating that the crystallinity and grain size of the samples significantly improved with increasing La doping, with the 5% La-doped sample exhibiting the best crystallinity.

### 3.2. Morphology

**Figure 2:**
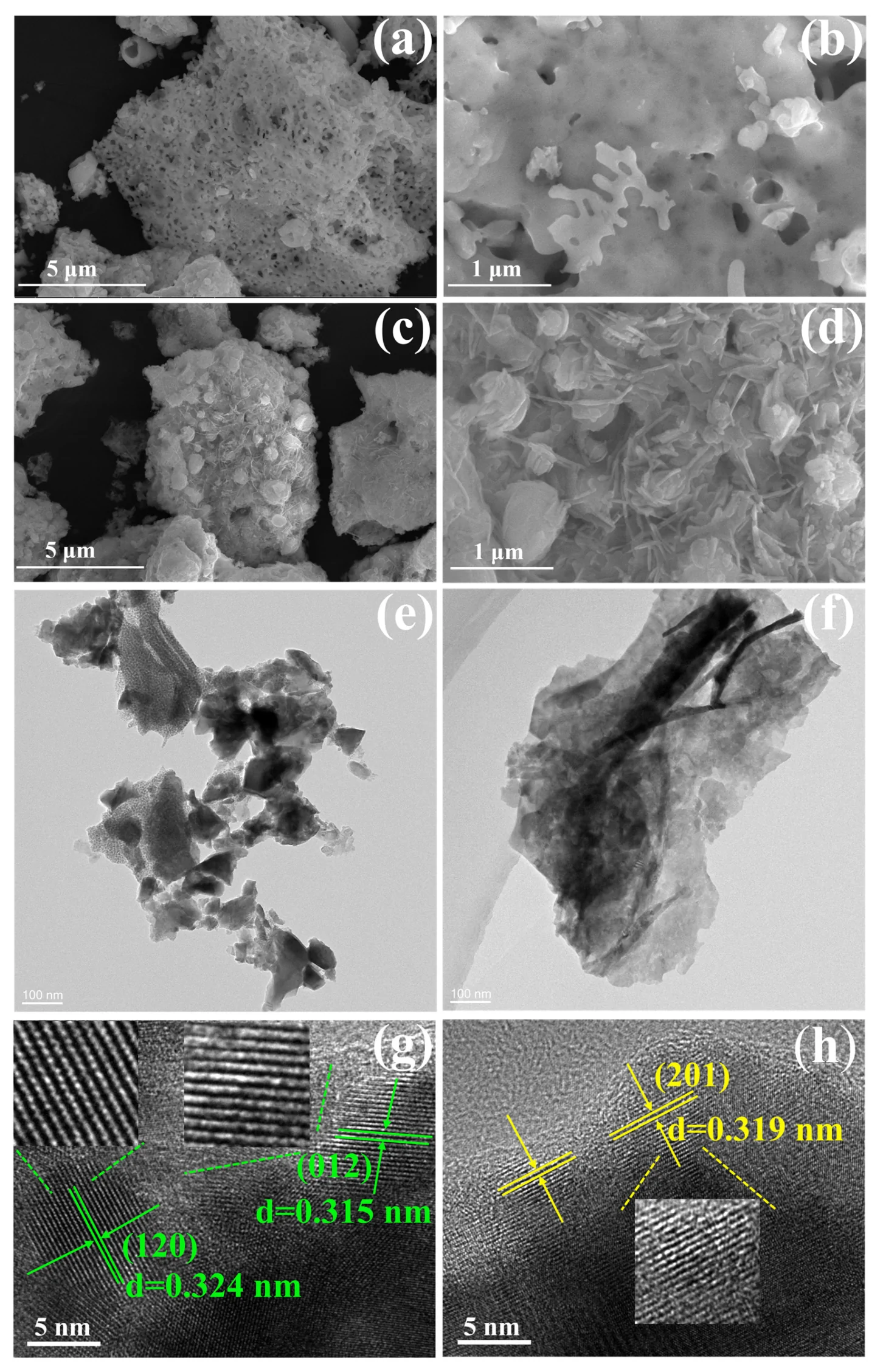
SEM images of Bi_2_O_3_ (**a**,**b**) and 4%La/Bi_2_O_3_ (**c**,**d**); TEM images of Bi_2_O_3_ (**e**) and 4%La/Bi_2_O_3_ (**f**); HRTEM images of Bi_2_O_3_ (**g**) and 4%La/Bi_2_O_3_ (**h**).

The morphology and internal microstructure of the pristine Bi_2_O_3_ and 4% La-doped Bi_2_O_3_ samples were systematically investigated using scanning electron microscopy (SEM) and transmission electron microscopy (TEM), while high-resolution TEM (HRTEM) was employed to examine the interfacial structural features. Figure 2a,b show the SEM morphologies of pure Bi_2_O_3_. At low magnification (Figure 2a), the sample primarily consists of micron-sized, sponge-like, porous agglomerates formed by the tight stacking and sintering of a large number of primary particles, with a rough surface; at ultra-high magnification (Figure 2b), nanoparticles are strongly aggregated to form a relatively dense layered structure, with localized areas of fused bonding visible, and irregular open pores distributed across the matrix surface [39]. However, in conjunction with the BET results (see below), it is evident that this rough surface did not translate into a high specific surface area (only 0.54 m^2^/g), indicating severe dense agglomeration among the particles, with a large number of potential pores buried within the agglomerates and thus unable to be exposed. The TEM image of pure Bi_2_O_3_ (Figure 2e) further shows that the grains are randomly stacked and tightly adhered to the layers, with the particles overlapping and agglomerating; there are no obvious monodisperse particles. After doping with 4% La, the SEM morphology of the sample changed significantly compared to that of pure Bi_2_O_3_ (Figure 2c,d). In Figure 2c, the product exhibits micrometer-sized irregular spherical agglomerates; the interior of these clusters is constructed from a complex interweaving of numerous nanoscale one-dimensional needle-like/fibrous structures and thin nanosheets, resulting in an overall loose and porous agglomerate. Figure 2d clearly shows that, under extremely high magnification, a large number of slender nanoneedles and nanofibers can be distinctly observed interlacing and overlapping with one another. These are accompanied by irregular, ultra-thin nanosheets and a small number of block-like grains, which cross-link to form a three-dimensional network pore structure [40]. The TEM image (Figure 2f) further confirms these structural features, revealing that the nanosheets interweave to form a three-dimensional network framework, with significantly improved dispersion compared to the pure sample. It can thus be seen that the microstructure of La-doped Bi_2_O_3_ exhibits a distinct regulatory effect, with the packing arrangement of particle aggregates transforming from a densely bonded state to a loosely interwoven three-dimensional framework structure, effectively suppressing dense particle agglomeration.

Based on the HRTEM images of the samples, in the Bi_2_O_3_ sample (Figure 2g), the interplanar spacings of 0.324 nm and 0.315 nm correspond to the (120) and (012) crystal planes, respectively, of the monoclinic phase α-Bi_2_O_3_ (PDF#76-1730). In the 4% La/Bi_2_O_3_ sample (Figure 2h), the crystal plane spacing of 0.319 nm corresponds to the (201) crystal plane of the tetragonal β-Bi_2_O_3_ phase (PDF#78-1793).

### 3.3. Chemical Structure and Nitrogen Adsorption Properties

**Figure 3:**
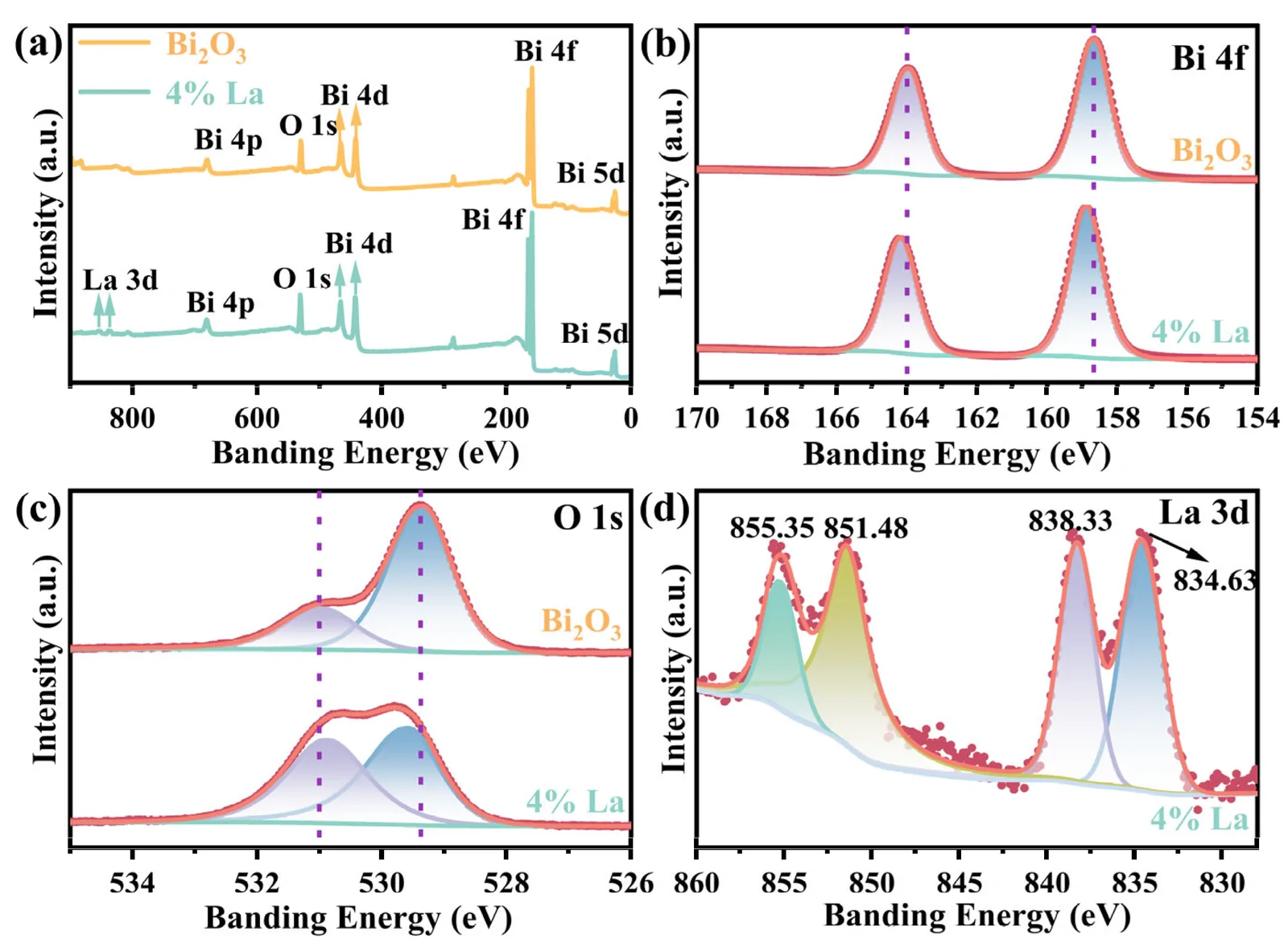
XPS spectra of Bi_2_O_3_ and 4%La/Bi_2_O_3_: (**a**) survey spectra, (**b**) Bi 4f, (**c**) O 1s, and (**d**) La 3d.

The surface chemical composition and valence states of the samples were characterized using XPS, with the binding energy positions of all peaks in Figure 3 corrected using the C 1s standard binding energy of 284.6 eV. The results (Figure 3a) show that the full spectrum of Bi_2_O_3_ exhibits characteristic peaks for the elements Bi, C, and O. In the full spectrum of 4% La/Bi_2_O_3_, in addition to the characteristic peaks for Bi, C, and O, characteristic peaks for La also appear, indicating that La has been successfully doped into Bi_2_O_3_.

In the high-resolution Bi 4f XPS spectrum shown in Figure 3b, the Bi 4f orbitals of both samples exhibit typical spin–orbit doublet features, corresponding to the Bi 4f_5/2_ and Bi 4f_7/2_ energy levels. In the pure Bi_2_O_3_ sample, the binding energies of Bi 4f_5/2_ and Bi 4f_7/2_ were 163.95 eV and 158.66 eV, respectively; after 4% La doping, both peaks shifted toward higher binding energies, moving to 164.20 eV and 158.88 eV, respectively. The overall increase in binding energy indicates that La doping alters the chemical environment surrounding the Bi atoms, reducing the electron density of the Bi atoms. This confirms that La^3+^ has successfully doped into the Bi_2_O_3_ lattice and formed Bi–O–La bonding, reflecting the effective modulation of the material’s electronic structure by doping [41,42,43].

The O 1s XPS spectrum of pure Bi_2_O_3_ (Figure 3c) shows two characteristic peaks: a low-binding-energy peak at 529.37 eV, attributed to lattice oxygen (Bi–O bond), and a high-binding-energy peak at 531.02 eV, corresponding to surface-adsorbed oxygen species. Following La doping, the lattice oxygen peak shifted positively to 529.60 eV (+0.23 eV), consistent with the trend of increased Bi 4f binding energy, confirming the lattice substitution of Bi^3+^ by La^3+^. The surface-adsorbed oxygen peak shifted only slightly to 530.88 eV (−0.14 eV); this change falls within the instrument’s margin of error and does not indicate a clear chemical shift. The increase in the integrated area of the surface-adsorbed oxygen peak after doping is primarily attributed to changes in the physicochemical state of the sample surface (such as an increase in specific surface area) and does not represent an increase in the concentration of lattice oxygen vacancies [44,45,46]. This inference is consistent with the experimental observation of an increased Bi 4f binding energy.

High-resolution La 3d XPS analysis was performed on a 4% La-doped Bi_2_O_3_ sample. The spectrum (Figure 3d) exhibits the typical bipodal structure of La^3+^ and its satellite peaks. The peaks at 834.63 eV and 838.33 eV are attributed to the main peak and satellite peak of La 3d_5/2_ and its satellite peak, respectively, while the peaks at 851.48 eV and 855.35 eV correspond to the main peak and satellite peak of La 3d_3/2_, respectively. The spin–orbit splitting between the two sets of peaks is approximately 16.8 eV. These spectral features are in complete agreement with the standard XPS data for La^3+^ [47,48,49], confirming that La has been successfully doped into the Bi_2_O_3_ lattice in the +3 oxidation state. This indicates that the chemical environment of La is stable after doping and that it exists in the material in ionic form.

**Figure 4:**
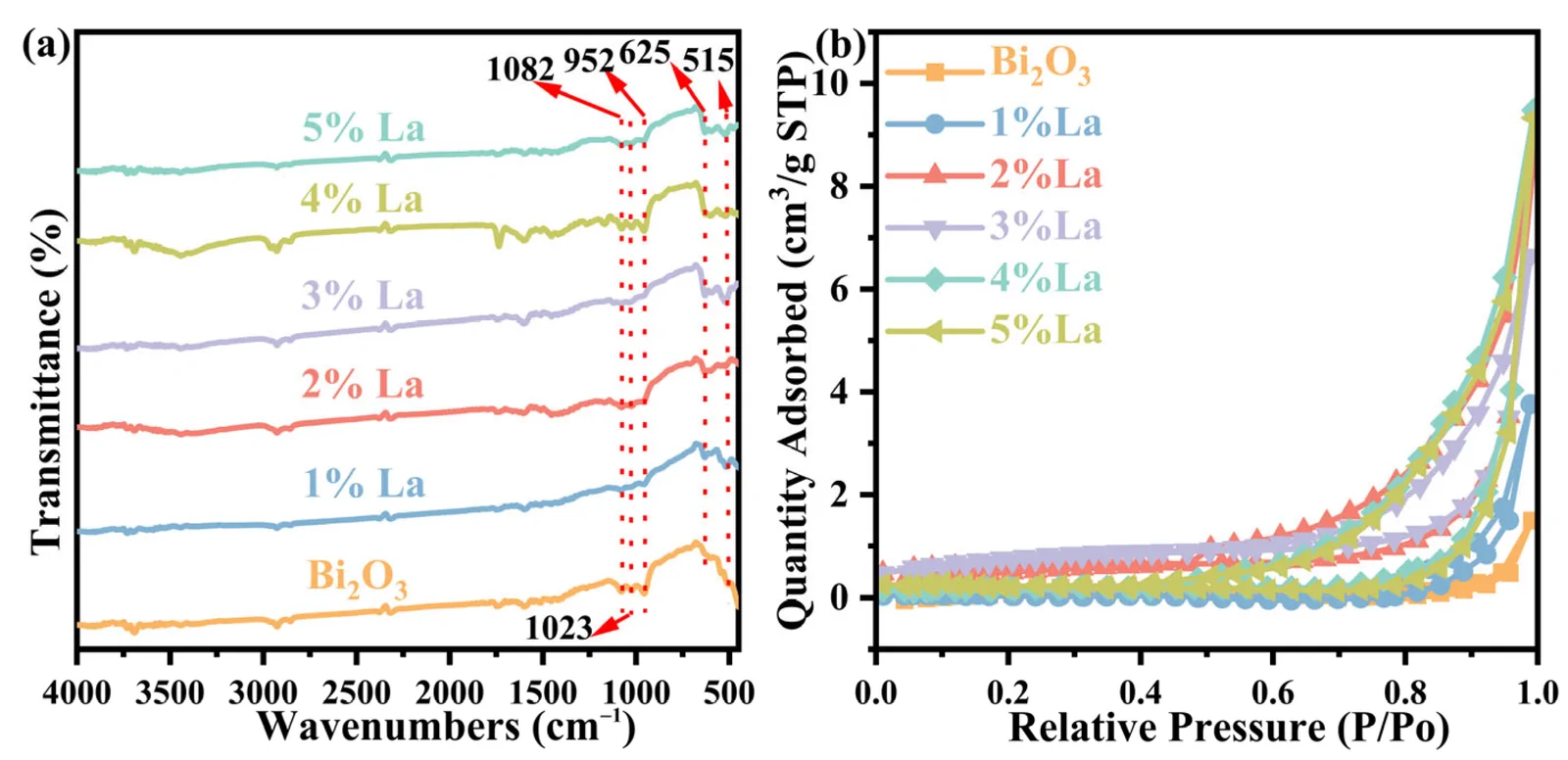
(**a**) FT-IR spectra of the photocatalysts and (**b**) N_d_ adsorption–desorption isotherms of the photocatalysts.

FTIR analysis was performed to identify the functional groups and chemical bonds in the synthesized photocatalyst and to determine its phase composition. Figure 4a shows the FTIR spectra of pure Bi_2_O_3_ and various La-doped samples. Free water adsorbed on the catalyst surface exhibits two characteristic absorption peaks in the ranges of approximately 1630 cm^−1^ and 3400–3700 cm^−1^, corresponding to the H–O–H bending vibration and the O–H stretching vibration, respectively. Additionally, the absorption peak near 1420 cm^−1^ can be attributed to the stretching vibration of the C=O bond in surface-adsorbed CO_2_. At 432 cm^−1^, only pure Bi_2_O_3_ exhibits a very sharp characteristic peak, attributed to the stretching vibration of the Bi–O bond in the BiO_6_ octahedra of α-Bi_2_O_3_ [50]; this peak disappears upon La doping, indicating the gradual disappearance of the α phase. Upon La doping, the samples exhibit distinct absorption peaks at 515 and 625 cm^−1^, originating from the stretching and bending vibrations of the Bi–O–Bi bonds in β-Bi_2_O_3_ [51]. The appearance of these β-Bi_2_O_3_ characteristic peaks indicates that La^3+^ doping induces an α → β phase transition, and the intensity of the β-phase characteristic peaks gradually increases with increasing La doping. At approximately 591 cm^−1^, the absorption peak becomes progressively more pronounced with increasing La^3+^ doping, possibly corresponding to the Bi–O stretching vibrations of non-bridging oxygen atoms in the distorted Bi–O polyhedra caused by La^3+^ doping [52]. The absorption peak at 591 cm^−1^ becomes progressively more pronounced as the La^3+^ doping concentration increases; these peaks are attributed to the Bi-O stretching vibrations of non-bridging oxygen atoms in the distorted Bi-O polyhedra caused by La^3+^ doping [53]. No characteristic peaks of La_2_O_3_ or other La-based compounds were observed in the FTIR spectrum, indicating that La^3+^ had successfully entered the Bi_2_O_3_ lattice to form a solid solution, without the formation of a distinct second phase. The above results confirm that pure Bi_2_O_3_ retains the α-phase structure, while the characteristic peaks of the β-phase begin to appear after La^3+^ doping, and the peak intensity increases with the increase in La^3+^ doping concentration. This is consistent with the XRD findings, namely that La^3+^ substitutes for Bi^3+^ to form a single-phase substitution solid solution, which does not disrupt the fundamental crystal framework of Bi_2_O_3_ but induces a phase transition from the monoclinic phase (α) to the tetragonal phase (β).

La ion doping can effectively modulate the microstructure of Bi_2_O_3_ and increase its specific surface area. Figure 4b shows the nitrogen adsorption–desorption isotherms and pore size distribution curves for pure Bi_2_O_3_ and samples with different La doping levels. As shown in Figure 4b, all samples exhibit typical Type IV isotherm characteristics and display a distinct H3 hysteresis loop within the relative pressure range of P/P_0_ ≈ 0.4–0.9, indicating the presence of slit-like mesoporous structures formed by particle aggregation in the prepared samples [54]. However, the nitrogen adsorption capacity of pure Bi_2_O_3_ is extremely low, with a BET specific surface area of only 0.54 m^2^/g. This stands in stark contrast to the sponge-like porous morphology observed by SEM, indicating that a large number of internal pores are in a closed or semi-closed state and are not effectively exposed at the surface, with severe dense sintering and agglomeration occurring between particles. As the La doping ratio was gradually increased from 0% to 5%, the BET specific surface area of the samples first increased and then decreased: the 1% La, 2% La, and 3% La-doped samples increased sequentially to 0.61, 0.88, and 1.70 m^2^/g; at 4% La doping, it reached a maximum of 2.68 m^2^/g, while the nitrogen saturation adsorption capacity was also at its highest, consistent with the three-dimensional interlaced network structure and improved dispersion observed by SEM/TEM; When the doping level was further increased to 5% La, the specific surface area decreased to 1.98 m^2^/g, which may be related to particle agglomeration and partial collapse of the pore structure caused by an excess of La. The above results indicate that an appropriate amount of La doping can effectively increase the material’s specific surface area by altering the particle packing arrangement and constructing a loose three–dimensional skeletal structure, thereby providing more exposed active sites for photocatalytic reactions and contributing to improved catalytic efficiency.

### 3.4. Spatial Charge Separation Capability

**Figure 5:**
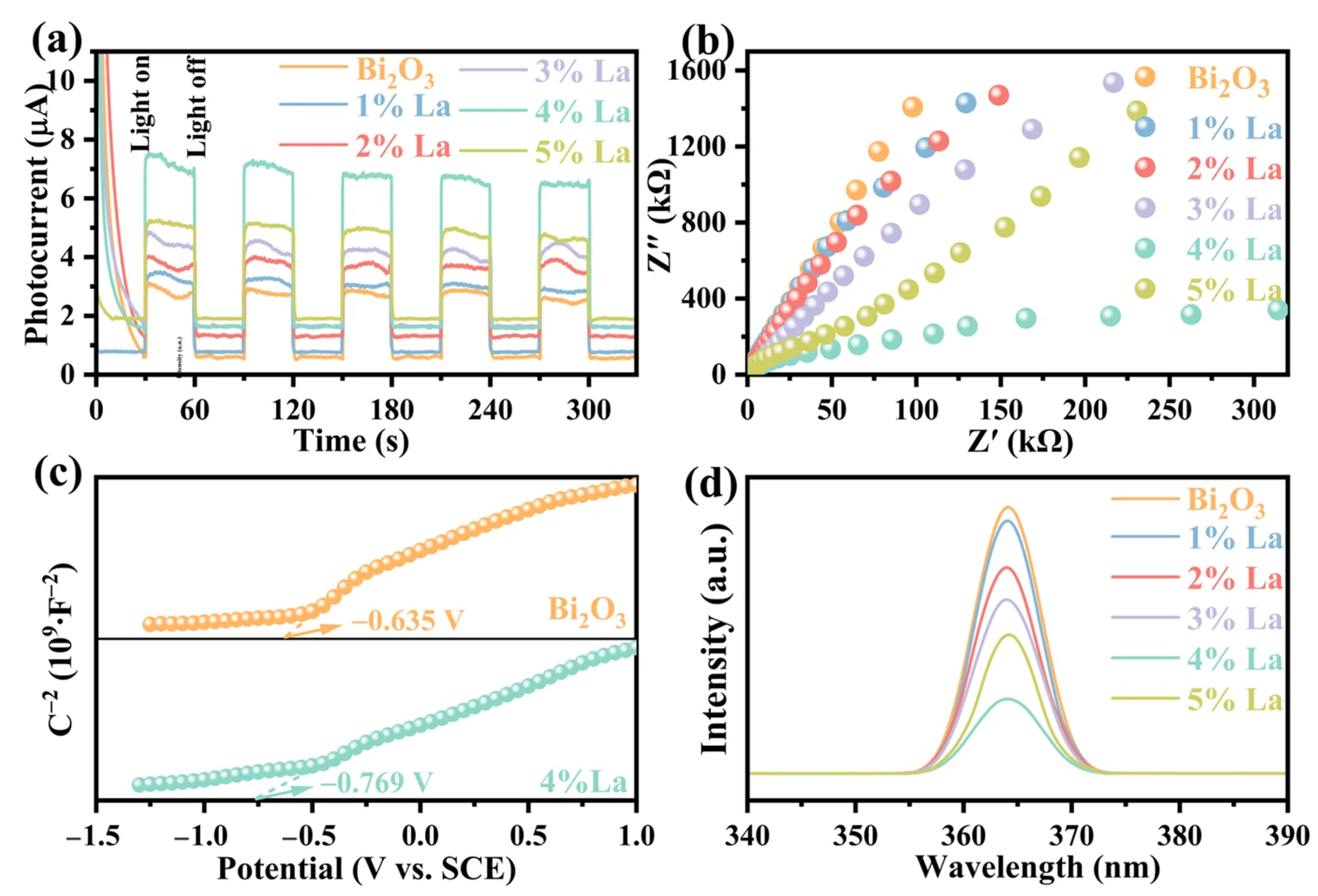
(**a**) Photocurrent response, (**b**) EIS Nyquist, (**c**) Mott–Schottky plots of Bi_2_O_3_ and 4% La/Bi_2_O_3_, and (**d**) photoluminescence emission spectra.

To evaluate the effect of La doping on the separation and migration behavior of photo-generated charge carriers in Bi_2_O_3_, transient photocurrent response tests were conducted on the prepared samples. As shown in Figure 5a, all samples exhibited rapid and stable photocurrent responses under multiple light–on/off cycles. Among them, the 4% La/Bi_2_O_3_ sample exhibited the highest photocurrent density, while pure Bi_2_O_3_ showed the weakest photocurrent response; the photocurrent values of the remaining doped samples fell between these two extremes, in descending order: 4% > 5% > 3% > 2% > 1% > pure Bi_2_O_3_. The excellent photoelectrochemical activity of the 4% sample can be attributed to the α→β phase transition induced by an optimal amount of La doping and the three-dimensional network framework structure, which effectively increases the specific surface area and provides abundant interfacial channels for the separation and transport of photo-generated charge carriers [55]. Simultaneously, the lattice substitution of Bi^3+^ by La^3+^ modulates the localized electronic structure, lowering the interfacial charge transfer barrier, thereby promoting the efficient separation of photo-generated electron–hole pairs and suppressing their recombination [56].

To further validate the above photocurrent results, electrochemical impedance spectroscopy (EIS) measurements were performed on the samples (Figure 5b) to investigate the effect of the La doping ratio on the charge transfer kinetics at the Bi_2_O_3_ interface. The Nyquist plot was fitted using the equivalent circuit model shown in the inset, yielding the solution resistance (Rs), the constant-phase element (CPE) at the electrolyte/electrode interface, and the charge transfer resistance (Rct). The Rct values for each sample followed the order 4% La/Bi_2_O_3_ < 5% La/Bi_2_O_3_ < 3% La/Bi_2_O_3_ < 2% La/Bi_2_O_3_ < 1% La/Bi_2_O_3_ < pure Bi_2_O_3_; this order is fully consistent with the transient photocurrent response. Among these, the 4% La/Bi_2_O_3_ sample exhibited the lowest Rct value, indicating the lowest interfacial charge transfer resistance and the most favorable conditions for the cross-interface migration of photo-generated carriers. The EIS results corroborate the photocurrent measurements, jointly confirming that an appropriate amount of La doping can effectively improve the interfacial charge transfer kinetics of Bi_2_O_3_ and promote the separation and migration of photo-generated carriers [57].

Mott–Schottky (M-S) testing was used to analyze the band structure and electrochemical characteristics of pure Bi_2_O_3_ and 4% La/Bi_2_O_3_. As shown in Figure 5c, the M-S curves of all samples exhibit a positive slope, indicating that the materials are all typical n-type semiconductors and that electrons are the primary charge carriers [58]. Using a saturated calomel electrode (SCE) as the reference electrode, the flat-band potentials of pure Bi_2_O_3_ and 4% La/Bi_2_O_3_ were −0.64 V and −0.77 V (vs. SCE), respectively; when converted to the standard hydrogen electrode (NHE), these correspond to −0.40 V and −0.53 V (vs. NHE) [59]. The flat-band potential of the 4% La/Bi_2_O_3_ sample exhibits a significant negative shift compared to the pure sample, indicating that La doping effectively shifts the Fermi level toward the conduction band, causing the bottom of the conduction band to shift to a more negative potential. The negative shift in the flat-band potential implies a reduction in the degree of band bending in the space charge layer and a narrowing of the space charge region, which facilitates the migration of photo-generated electrons toward the material surface and suppresses bulk electron–hole recombination. At the same time, the more negative position of the conduction band bottom confers stronger reducing power to the photo-generated electrons [60].

By characterizing the photoluminescence (PL) spectra, we investigated the effect of La doping on the separation and recombination behavior of photo-generated carriers in Bi_2_O_3_. A lower fluorescence intensity indicates a slower electron–hole pair recombination rate, meaning that more effective carriers participate in interfacial redox reactions [61]. This explains, from the perspective of carrier dynamics, the intrinsic mechanism by which doping enhances photocatalytic performance. A specific amount of the sample was dispersed in pure water for fluorescence performance testing; the setup is shown in Figure 5d. La doping effectively quenches the fluorescence emission of Bi_2_O_3_ and suppresses the rapid recombination of photo-generated electron–hole pairs. This is attributed to the modulation of the localized electronic structure caused by the lattice substitution of La^3+^ for Bi^3+^, which facilitates the effective separation of photo-generated carriers. As the La doping ratio increased, the carrier separation efficiency first increased and then decreased slightly; the sample doped with 4% La exhibited the lowest fluorescence intensity, which was fully consistent with its highest photocurrent density and lowest charge transfer resistance.

**Figure 6:**
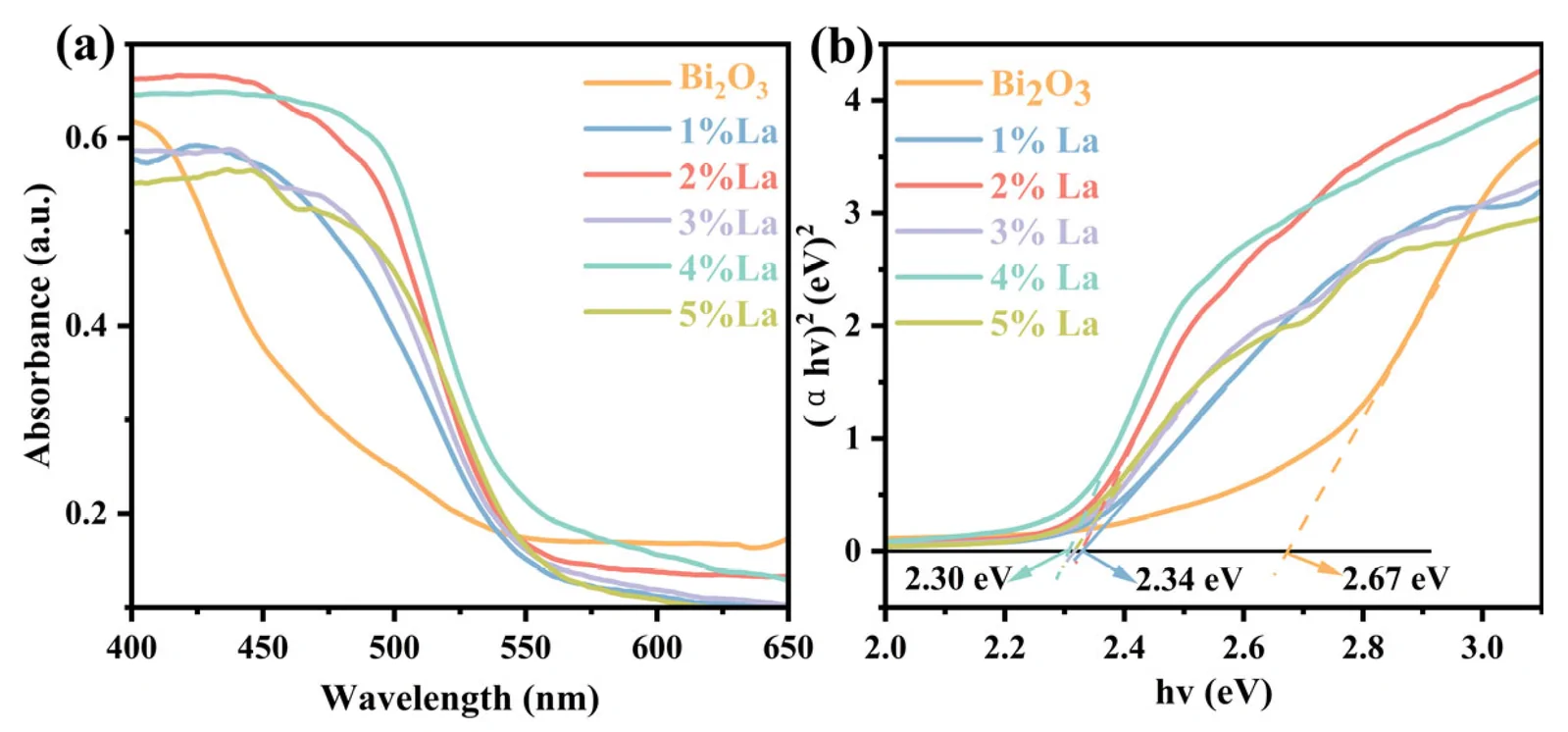
(**a**) UV-vis DRS, and (**b**) corresponding Tauc plots of diverse photocatalysts.

Optical properties and band structure are key factors in evaluating the photocatalytic performance of semiconductor materials. According to the UV-Vis DRS results (Figure 6a), the absorption edge of pure Bi_2_O_3_ undergoes a significant red shift upon La doping, indicating that the introduction of La effectively broadens the material’s optical response range. However, the positions of the absorption edges among samples with different La doping levels do not differ significantly, which is consistent with the XRD results: all La-doped samples consist of the pure β phase, and their similar crystal structures result in closely matching optical absorption characteristics. Analysis of the Tauc plot [62] further revealed the trend in bandgap changes (Figure 6b): the bandgap of pure Bi_2_O_3_ was 2.67 eV, while that of all La-doped samples narrowed significantly to the range of 2.30–2.34 eV. Among them, the 4% La-doped sample exhibited the smallest bandgap at 2.30 eV. The significant narrowing of the bandgap may be attributed to orbital hybridization between the 4f orbitals of La^3+^ and the band structure of Bi_2_O_3_, as well as to the electronic restructuring accompanying the La^3+^ substitution-induced α → β phase transition. Although the extent of bandgap narrowing varies little across different La doping concentrations, compared to the undoped sample, the marked reduction in the bandgap facilitates the generation of more photo-generated carriers under equivalent illumination conditions [63]. Combined with the PL and EIS results, the 4% sample exhibits both the lowest carrier recombination rate and the smallest interfacial transfer resistance, and the synergistic effect of these two factors enables it to demonstrate a more efficient utilization of visible light.

### 3.5. Photocatalytic Performances and Stability

**Figure 7:**
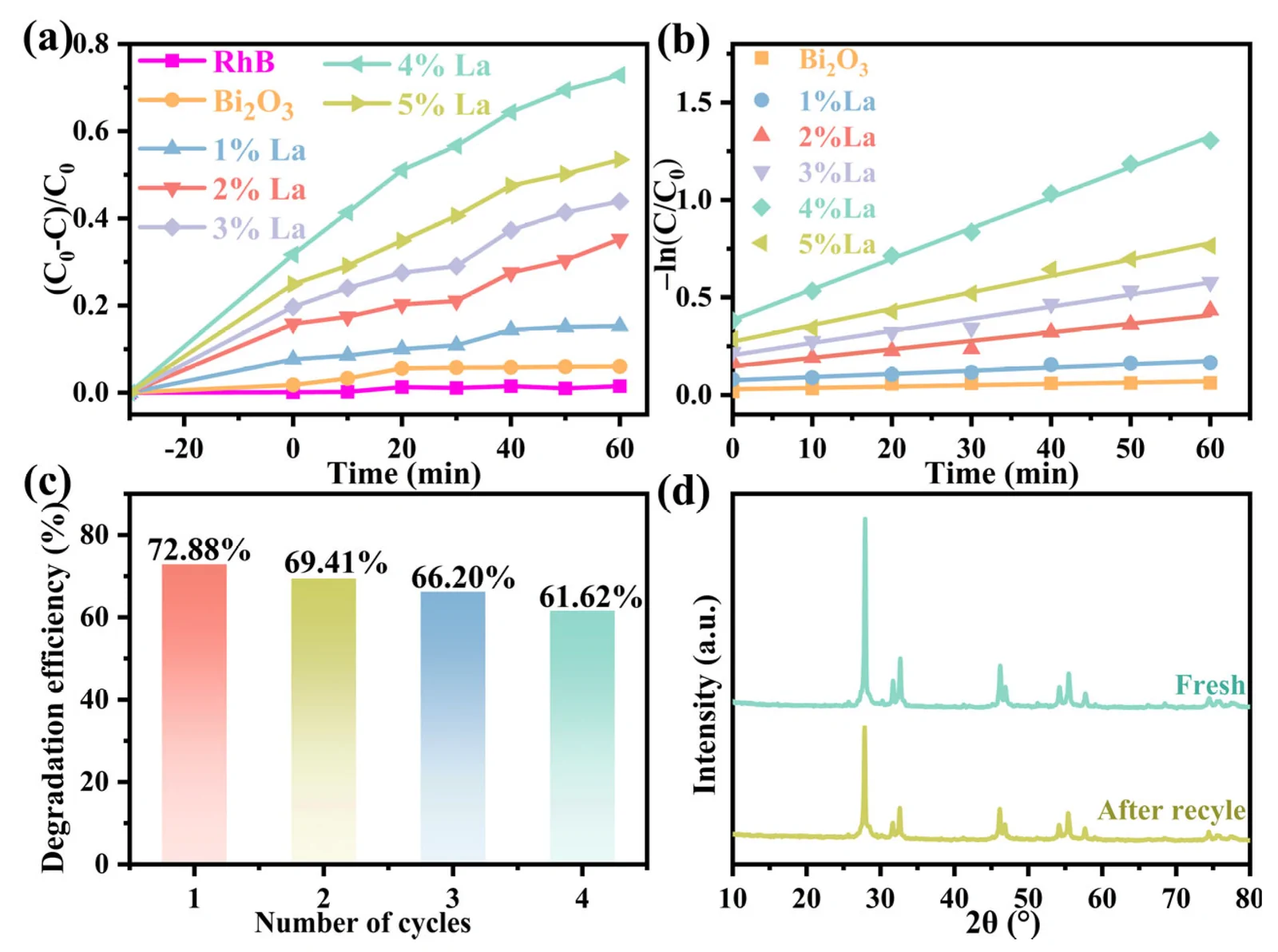
(**a**) Visible-light-driven RhB degradation curves of various samples under dark adsorption and visible-light irradiation; (**b**) corresponding pseudo-first-order kinetic fitting plots for different samples; (**c**) recycling stability tests of pristine Bi_2_O_3_ and 4% La/Bi_2_O_3_; (**d**) XRD patterns of Bi_2_O_3_ and 4% La/Bi_2_O_3_ before and after cyclic photocatalytic reactions.

To verify the photocatalytic performance of the samples, photocatalytic degradation experiments using RhB were conducted under visible-light irradiation. A xenon lamp was used to simulate sunlight; prior to turning on the lamp, the samples were stirred for 30 min in the dark to achieve adsorption–desorption equilibrium. As shown in Figure 7a, in the absence of a catalyst, RhB exhibited good stability and showed almost no degradation under visible light, indicating that the photolysis of RhB by visible light itself is negligible. In the presence of a catalyst, RhB underwent significant degradation. After 60 min of illumination, pure Bi_2_O_3_ exhibited the lowest RhB degradation rate, at only 6.04%; the photocatalytic performance of the La-doped samples was significantly improved, with the 4% La/Bi_2_O_3_ sample showing the best performance, achieving a degradation efficiency of 72.88%. The RhB degradation processes for both pure Bi_2_O_3_ and the various La-doped samples followed a quasi-first-order kinetic model (Figure 7b) [64]. The quasi-first-order reaction rate constants for Bi_2_O_3_, 1%La, 2%La, 3%La, 4%La, and 5%La/Bi_2_O_3_ were 0.000680, 0.001638, 0.004370, 0.006210, 0.015690, and 0.008420 min^−1^, respectively. Clearly, 4%La/Bi_2_O_3_ exhibits the optimal photocatalytic performance, with a rate constant approximately 23 times that of pure Bi_2_O_3_. This result is highly consistent with the aforementioned characterization conclusions: moderate La doping synergistically enhances the visible-light photocatalytic activity of Bi_2_O_3_ by inducing an α → β phase transition, increasing the specific surface area, optimizing the band structure, and promoting the separation and migration of photo-generated charge carriers; whereas the decline in performance observed in the 5% sample is attributed to particle agglomeration and a decrease in specific surface area caused by excessive doping.

To clarify the performance positioning of the 4% La/Bi_2_O_3_ prepared in this study within similar systems, representative modified Bi_2_O_3_ photocatalysts from the literature were selected for a comparative analysis (Table 1). Zhou et al. [65] reported that the rate constant for RhB on the Bi_2_O_3_/carbon paper composite was only 0.00372 min^−1^ (approximately 58.3%, 120 min), while Hu et al. [66] reported a rate constant of 0.00798 min^−1^ for tetracycline on the CeO_2_/Bi_2_O_3_ heterojunction (79.93%, 180 min), and the rate constant for methylen blue reported by Xia et al. [67] for the Bi_2_O_3_/WO_3_ p-n heterojunction was 0.01000 min^−1^ (56.3%, 60 min). Furthermore, although Xue et al. [39] did not provide the first-order reaction rate constant for La/Ce co-doped Bi_2_O_3_ in their paper, the degradation rate of Acid Orange II reached 85.93% within 100 min. The results show that the sample prepared in this study exhibits outstanding degradation efficiency and reaction rates for RhB under visible-light irradiation. Its overall photocatalytic performance surpasses most of the values reported in the literature, and the synthesis process is simpler, demonstrating good practical potential.

The recyclability of photocatalytic materials is of great significance for their practical applications. To this end, cyclic degradation experiments were conducted using 4% La/Bi_2_O_3_. Figure 7c visually illustrates the trend in the catalyst’s efficiency during four consecutive cycles of RhB degradation. The degradation efficiency in the first cycle was 72.88%; as the number of cycles increased, the efficiency exhibited a steady, gradual decline without any abrupt drop. After four cycles of reuse, the material retained 61.62% of its degradation capacity, indicating that this catalyst possesses excellent recyclability. The slight decline in efficiency may be attributed to contaminants adsorbed on the sample surface that were not completely desorbed, thereby covering some of the active sites [68]. At the same time, repeated centrifugation and washing processes may also cause mechanical loss of trace amounts of the catalyst.

The structural stability of the photocatalyst before and after use is equally critical. Figure 7d compares the XRD patterns of the pristine catalyst and the recycled catalyst; the 2θ positions and relative intensities of all characteristic diffraction peaks in both samples show a high degree of consistency. No new impurity diffraction peaks appeared, and the original characteristic peaks did not shift or disappear. These results indicate that repeated cyclic reactions did not cause structural damage, such as phase transformations or a significant decrease in crystallinity, confirming that 4% La/Bi_2_O_3_ possesses excellent structural stability.

The above cycling experiments and structural stability analysis collectively demonstrate that, after multiple cycles of reuse, this catalyst exhibits no significant deterioration in either its photocatalytic performance or crystal structure. It possesses good reusability and holds potential for application in practical water pollution treatment, thereby helping to reduce operating costs.

### 3.6. Photocatalytic Mechanism

**Figure 8:**
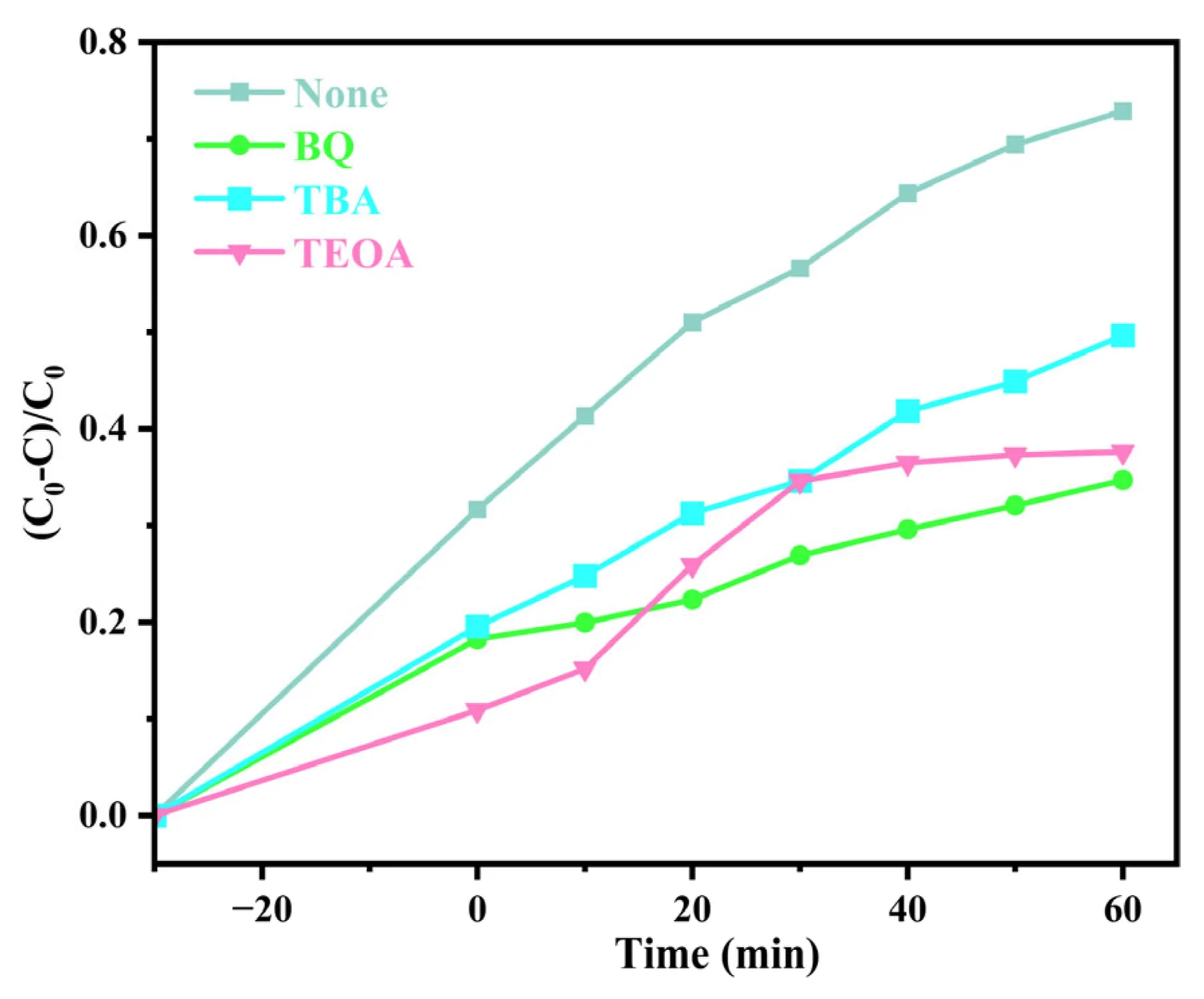
Visible-light-driven RhB photodegradation over 4% La/Bi_2_O_3_ with various radical scavengers, including BQ for ·O_2_^−^, TBA for ·OH and TEOA for h^+^.

Radical trapping experiments were conducted to investigate the primary active radical species involved in the degradation of RhB by 4% La/Bi_2_O_3_ under visible light. In the experiments, anthraquinone (BQ), tert-butyl alcohol (TBA), and triethanolamine (TEOA) were used as scavengers for the superoxide radical (·O_2_^−^), hydroxyl radical (·OH), and holes (h^+^), respectively. As shown in Figure 8, the photocatalytic degradation rates of the samples decreased to varying degrees upon the addition of different scavengers, indicating that all three reactive species contribute to the photocatalytic reaction. Notably, the addition of BQ and TEOA resulted in very significant inhibition of the degradation reaction, whereas the addition of TBA led to a relatively weaker inhibition of the RhB degradation rate. Based on these results, the order of contribution of the active species can be determined as superoxide radical (·O_2_^−^) > hole (h^+^) > hydroxyl radical (·OH). This indicates that the photocatalytic reaction of this catalyst primarily relies on the strong oxidizing action of the superoxide radical (·O_2_^−^), while the role of the hole (h^+^) is also significant, and the hydroxyl radical (·OH) plays only a supporting role.

The equivalent substitution of Bi^3+^ in the lattice with an appropriate amount of La^3+^ (4%) not only induces the complete transformation of pure α-Bi_2_O_3_ into the tetragonal β-Bi_2_O_3_ phase, but also induces grain rearrangement and changes in particle packing, thereby constructing a three-dimensional network mesoporous structure. This increases the specific surface area to the highest value in the series and significantly increases the number of active sites on the surface. La doping narrows the bandgap to 2.30 eV, broadening the visible-light response range; the modulation of the local electronic structure caused by La^3+^ substitution effectively reduces the interfacial charge transfer resistance and increases the photocurrent density, thereby significantly suppressing the recombination of photo-generated electron−hole pairs.

**Figure 9:**
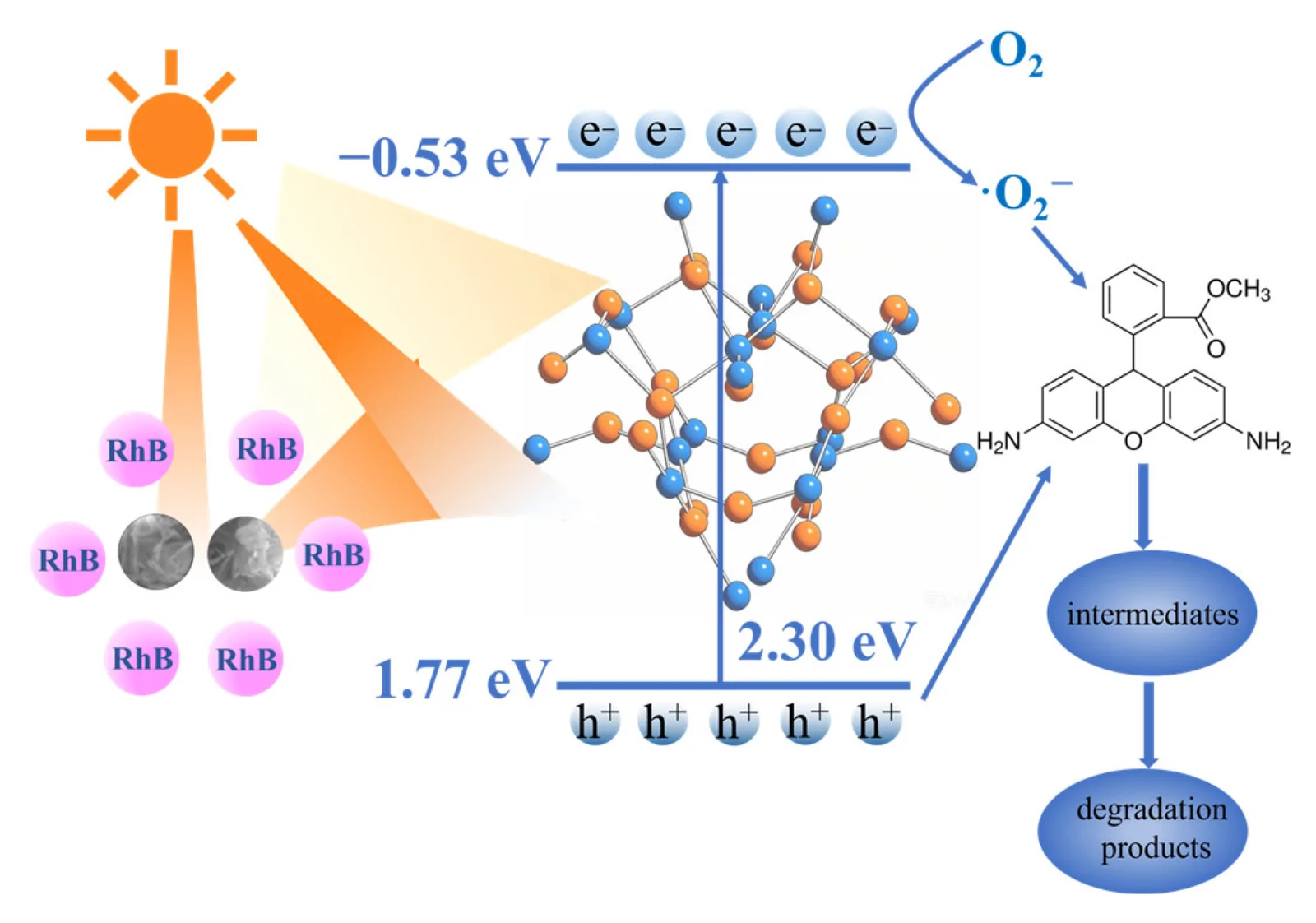
Possible mechanism of photocatalytic RhB degradation over 4% La/Bi_2_O_3_ heterojunction under visible light.

Under visible-light irradiation, valence band electrons undergo an excited transition to the conduction band, generating photo-generated electrons (e^−^) and holes (h^+^). Since the material is an n-type semiconductor with a conduction band bottom potential of −0.53 V (vs. NHE)—which is more negative than the redox potential of O_2_/·O_2_^−^ (−0.33 V vs. NHE)—photo-generated electrons can rapidly reduce dissolved oxygen to superoxide anion (·O_2_^−^), making it the dominant active species responsible for the degradation of RhB [69,70,71]. Meanwhile, the valence band edge potential is +1.77 V (vs. NHE), which is lower than the oxidation potentials of ·OH/H_2_O (+2.68 V) and OH^−^/·OH (+1.99 V). Holes cannot directly oxidize water or hydroxide ions to form hydroxyl radicals; they can only directly oxidize RhB molecules adsorbed on the catalyst surface. Only trace amounts of ·OH are generated in the system via chain reactions involving ·O_2_^−^, playing a supplementary role in degradation [72,73]. Radical trapping experiments (Figure 8) indicate that the order of contribution of reactive species is ·O_2_^−^ > h^+^ > ·OH. These three species synergistically attack the RhB molecule, gradually destroying its conjugated chromophore structure. As shown in Figure 7a, as the illumination time increases, the intensity of RhB’s characteristic absorption peak at 554 nm gradually weakens, indicating that its chromophore structure has been effectively destroyed. It should be noted that this study primarily monitored the removal efficiency and reaction kinetics of the RhB parent molecule via UV-visible spectroscopy. According to literature reports [74], during the photocatalytic oxidation process, RhB undergoes continuous attack by reactive species, followed by steps such as deethylation, chromophore cleavage, and aromatic ring opening, ultimately being gradually mineralized into CO_2_ and H_2_O. Based on the above analysis, Figure 9 illustrates the possible photocatalytic mechanism for the degradation of RhB by 4% La/Bi_2_O_3_ under visible light. The photocatalytic degradation process studied in this research can be described by the following chemical reaction equations:

4%La/Bi_2_O_3_ + hν → 4% La/Bi_2_O_3_ (e^−^ + h^+^) (1)

e^−^ + O_2_ → ·O_2_^−^ (2)

·O_2_^−^ + RhB → intermediates → degradation products (3)

h^+^ + RhB → intermediates → degradation products (4)

## 4. Conclusions

This work systematically investigated the effects of different La doping levels on the phase structure, microstructure, band structure, and photo-generated carrier dynamics of Bi_2_O_3_. The main conclusions are as follows:

(1) For the first time, a threshold effect of La doping inducing a complete phase transition in Bi_2_O_3_ was revealed; as little as 1% La is sufficient to trigger a complete α → β transition, simultaneously narrowing the bandgap from 2.67 eV to 2.30 eV, which effectively broadens the visible-light response range and suppresses carrier recombination.

(2) La doping transformed the material’s morphology from dense agglomerates to a three-dimensional porous network structure, increasing the specific surface area from 0.54 to 2.68 m^2^/g and providing more exposed active sites for photocatalytic reactions.

(3) The 4% La/Bi_2_O_3_ sample exhibited optimal photocatalytic performance, achieving a RhB degradation rate of 72.88% within 60 min, with a reaction rate constant 23 times that of pure Bi_2_O_3_. Furthermore, ·O_2_^−^ was the dominant active species in the degradation process, followed by h^+^ and ·OH.

La/Bi_2_O_3_ materials demonstrate good application potential in the field of wastewater treatment, particularly for the photocatalytic degradation of organic pollutants under visible light. Their excellent reusability and structural stability further support their feasibility in sustainable water purification. With further optimization, this material can also be extended to the degradation of antibiotics and other emerging pollutants, as well as to the field of solar-driven environmental remediation.

## Figures and Tables

**Table 1:** Comparison of the photocatalytic performance of 4% La/Bi_2_O_3_ in this study with prior research.

Catalyst	k (min^−1^)	Pollutant	Degradation Efficiency @ Time	Reference
4% La/Bi_2_O_3_	0.01569	RhB	72.88% @ 60 min	This work
Bi_2_O_3_/CP	0.00372	RhB	about 58.3% @ 120 min	[65]
CeO_2_/Bi_2_O_3_	0.00798	TC	79.93%@ 180 min	[66]
Bi_2_O_3_/WO_3_	0.01000	MB	56.3% @ 60 min	[67]
3%La/1%Ce/Bi_2_O_3_	—	acid Orange	85.93% @ 100 min	[39]

— = not listed.

## Data Availability

All data are available to download within the database.

## Supplementary Materials

The following supporting information can be downloaded at: https://www.mdpi.com/article/10.3390/nano16161025/s1, Figure S1: Photos of the powdered product; Figure S2: Schematic diagram of a photocatalytic reactor; Figure S3: Schematic diagram of an photoelectrochemical measurement system.
