# Supplementary material for: La-Induced Phase Transformation and Band Structure Modulation of Bi2O3 for Enhanced Visible-Light Photocatalytic Degradation of Rhodamine B
Source: Nanomaterials (Basel). 2026 Aug 18;16(16):1025. doi: 10.3390/nano16161025 (PMC13516286; doi:10.3390/nano16161025)
Supplement: Supplementary file 1 [file nanomaterials-16-01025-s001.zip › nanomaterials-4509202-supplementary.pdf]

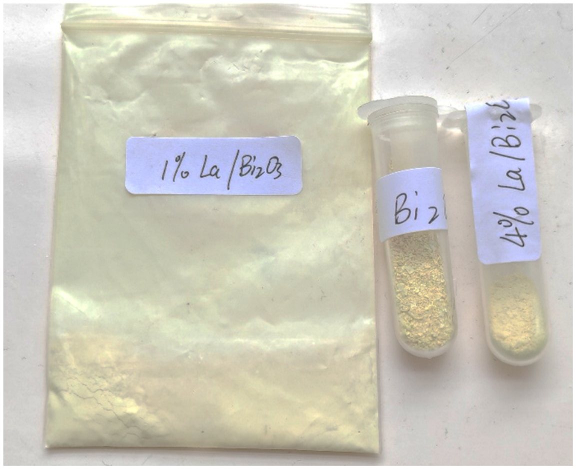

Figure S1. Photos of the powdered product.

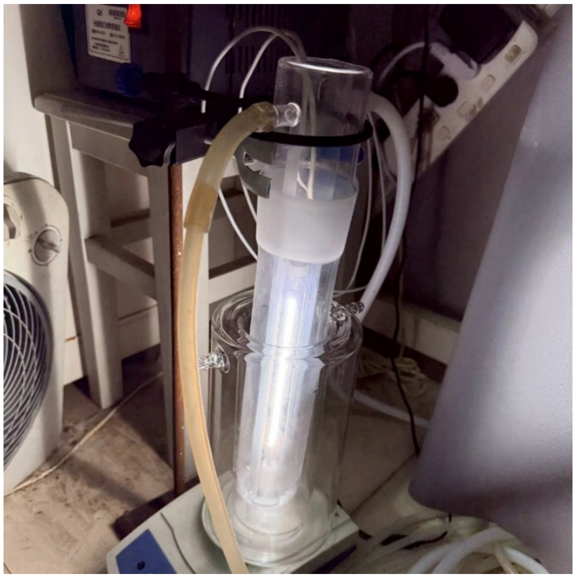

Figure S2. Schematic diagram of a photocatalytic reactor.

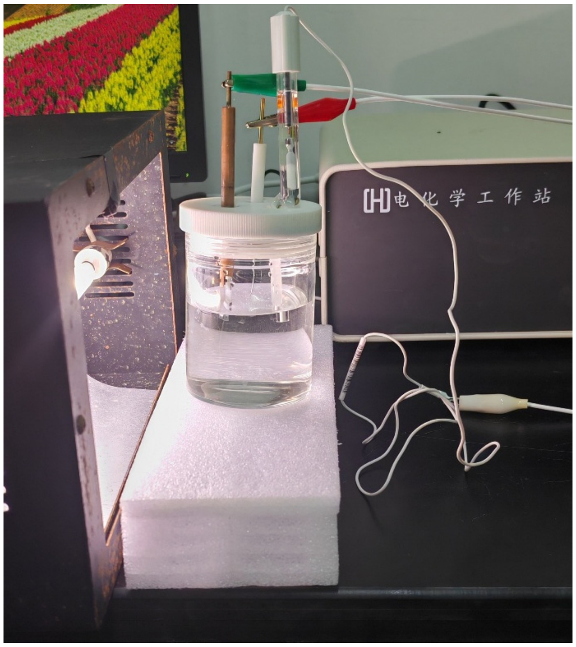

Figure S3. Schematic diagram of an photoelectrochemical measurement system.
